# GPR84 potently inhibits osteoclastogenesis and alleviates osteolysis in bone metastasis of colorectal cancer

**DOI:** 10.1186/s13018-022-03473-y

**Published:** 2023-01-02

**Authors:** Li Jian, Long Shi-wei, Jing Dan, Wu Juan, Zheng Wei

**Affiliations:** 1 Department of Orthopedics, General Hospital of Western Theater Command, Rongdu Avenue No. 270, Chengdu, 610000 People’s Republic of China; 2 Chengdu Medical College, Rongdu Avenue No. 601, Chengdu, 610000 People’s Republic of China; 3 Department of Pharmacy, General Hospital of Western Theater Command, Rongdu Avenue No. 270, Chengdu, 610000 People’s Republic of China

**Keywords:** GPR84, BMMs, MAPK pathway, Bone metastasis, Colorectal cancer

## Abstract

The expression of GPR84 in bone marrow-derived monocytes/macrophages (BMMs) can inhibit osteoclast formation; however, its role in bone metastasis of colorectal cancer (CRC) is still unknown. To investigate the effects of GPR84 on bone metastasis of CRC, the murine CRC cell line MC-38 was injected into tibial bone marrow. We found that the expression of GPR84 in BMMs was gradually downregulated during bone metastasis of CRC, and the activation of GPR84 significantly prevented osteoclastogenesis in the tumor microenvironment. Mechanistically, the MAPK pathway mediated the effects of GPR84 on osteoclast formation. Moreover, we found that IL-11 at least partly inhibited the expression of GPR84 in the tumor microenvironment through the inactivation of STAT1. Additionally, activation of GPR84 could prevent osteolysis during bone metastasis of CRC. Our results suggest that CRC cells downregulate the expression of GPR84 in BMMs to promote osteoclastogenesis in an IL-11-dependent manner. Thus, GPR84 could be a potential therapeutic target to attenuate bone destruction induced by CRC metastasis.

## Introduction

Bone is one of the most frequent metastatic sites for advanced colorectal cancer (CRC) [1]. Bone metastasis often contributes to skeletal-related events (SREs) [2, 3]. Pathological fracture, pain and hypercalcemia are commonly observed in patients with cancer bone metastasis [2]. Importantly, bone metastasis not only impacts the quality of life of patients but also reduces their overall survival.

In most situations, the skeletal pathological changes after bone metastasis can be classified as osteolytic or osteoblastic [4, 5]. Interestingly, osteolysis can occur after bone metastasis of several cancers with high incidences, such as lung cancer, breast cancer (BC) or CRC [6, 7]. Treatment of bone metastasis could prevent disease progression and alleviate symptoms.

Bone is a dynamic tissue. Two dominant cell types, osteoblasts and osteoclasts (OCs), are responsible for bone remodeling and the maintenance of bone homeostasis [8]. During cancer bone metastasis, osteoblasts or osteoclasts are aberrantly activated. The balance between bone formation and resorption is broken, leading to sclerosis or lysis [9]. In osteolytic metastasis, abnormally activated osteoclasts (OCs), as well as their precursors (OCPs), contribute to bone resorption [5]. Osteoclasts are large multinucleated cells derived from the monocyte/macrophage lineage. Osteoclast differentiation often requires RANKL and M-CSF. In a nontumorigenic environment, these factors can be derived from osteoblasts, osteocytes and immune cells [10]. However, cancer cells can produce abundant osteoclastogenic factors during bone metastasis to promote osteoclast formation and bone resorption in a RANKL-dependent or RANKL-independent manner [11]. A previous study demonstrated that targeting tumor cell-derived factors efficiently prevents osteoclast formation and could be a potential target for bone resorption in bone metastasis [11].

G protein-coupled receptors (GPRs) are a large group of membrane receptors that play critical roles in mediating cellular responses to external stimuli. GPRs can be stabilized by ligands or agonists to interact with a heterotrimeric G protein, thus mediating downstream signaling activity. GPRs play a role in the interaction between cancer cells and other cells and regulate metastasis [12]. Several GPR family members have been reported to be involved in tumor metastasis [13–15]. In bone metastasis, ovarian cancer GPR1 mediates communication between cancer cells and osteoblast-like cells [16]. In addition, GPR65 is downregulated in OCPs during bone metastasis of CRC by miR-7062-5p [17]. Moreover, GPR65/ADGRG1 is upregulated in 4T1 cells, a BC cell line, and in bone metastatic foci of patients with BC [18]. Interestingly, overexpression of GPR84 was reported to prevent osteoclast formation and bone resorption [19]. Notably, GPR84 is highly expressed in monocytes/macrophages [20], which are the source of osteoclasts. However, the role of GPR84 in bone metastasis of CRC is still unclear.

Herein, we present that the expression of GPR84 in OCPs is downregulated in bone metastasis of CRC. Activation of GPR84 efficiently prevents osteoclast formation as well as bone resorption by targeting the MAPK pathway. Moreover, we identified that IL-11 derived from cancer cells at least partially inhibits the expression of GPR84 in OCPs by inactivating STAT1.

## Materials and methods

### Animal experiments

All animal experiments and procedures were approved by the Animal Care and Use Committee of General Hospital of Western Theater. C57BL/6 wild-type mice (6–8 wk old) were utilized for the in vivo experiments. For bone metastasis studies, 1.0 × 10^6^ MC-38 cells were suspended in PBS and injected into the tibias of anesthetized mice. To study responses to 6-OAU, mice were orally treated with 6-OAU (2 μM, Selleck) dissolved in PBS for one week. In some experiments, mouse tibial bone marrow was injected with 2 μg of recombinant mouse IL-11 (BioLegend) once a week or 10 μg of IL-11 Nab (R&D) twice a week until harvest, while the control group was administered PBS. μCT analysis was performed at specific timepoints. After 3 weeks, the hindlimbs were removed and fixed in 10% neutral-buffered formalin for TRAP or immunohistochemical staining.

### Cancer cell culture and conditioned medium collection

MC-38 cells were plated in Matrigel-coated tissue culture plates and cultured with growth medium (GM) containing RPMI 1640 supplemented with 10% FBS and 1% penicillin‒streptomycin. The culture medium was changed every 2 d. Conditioned medium (CM) was made of RPMI. For collection, cancer cells were washed 3 times with PBS after reaching 80% confluence and cultured in CM for 24 h. The CM was collected and centrifuged to remove debris and cells.

### Isolation of BMMs from mice and cell culture

Bone marrow was rinsed out from the tibias of the animals. The cells were cultured in α-minimal essential medium (MEM) containing 10% FBS and 1% penicillin‒streptomycin with M-CSF (50 ng/ml) for 24 h. Then, suspended cells were collected and transferred into a new culture flask to culture for 24 h. The adherent cells were cultured in α-minimal essential medium (MEM) containing 10% FBS and 1% penicillin‒streptomycin with M-CSF (50 ng/ml). The culture medium was changed every three days.

### Osteoclast differentiation and quantification

Following euthanasia, the long bones of the animals were isolated. Bone marrow was flushed with sterile PBS. Red blood cells (RBCs) were lysed using RBC lysis buffer (Solarbio). Equal cell numbers were cultured overnight in 50 ng/mL M-CSF (BioLegend) for 24 h to obtain bone marrow-derived monocytes/macrophages (BMMs). The suspension cells were cultured in α-Minimal Essential Medium (αMEM) containing 10% FBS and 1% penicillin‒streptomycin solution with M-CSF (50 ng/mL) and RANKL (100 ng/mL). The CM treatments were 1:1 with αMEM with 10% FBS. To detect the effects of cytokines on the expression of GPR84 in BMMs, CTGF (50 ng/mL), IL-11 (10 ng/mL), PTHrP (100 nM) and EGF (10 ng/mL) were added. For treatment with agonists, cells were seeded in 24-well plates and treated with 6-OAU (200 nM) for 6 h. To activate the MAPK pathway, 50 μM tert-butylhydroquinone (t-BHQ) or 10 μM anisomycin was added to the culture medium for 6 h. In some experiments, cells were transfected with pCMV6-GPR84 for 24 h. Then, osteoclast differentiation was carried out for two to four days, with the differentiation medium being replaced on day three. For tartrate-resistant acid phosphatase (TRAP) staining, cells were fixed in 4% paraformaldehyde (PFA) for 30 min and then stained with TRAP staining solution (Wako, Japan) according to the manufacturers’ instructions.

### Proliferation assay

BMMs were cultured with M-CSF (50 ng/mL) and treated with 6-OAU (200 nM) for 6 h. Cells were labeled with BrdU (10 μM, BioLegend) for 2 h before sample collection. The samples were fixed with 2% PFA and permeated with 1% Triton X-100 for 15 min at 37 °C. After washing with PBS three times, the samples were incubated with anti-BrdU conjugated with FITC (BioLegend) for 30 min in the dark. BrdU incorporation was detected by a BD FACSVerse flow cytometer (BD Biosciences).

### Flow cytometry analysis

BMMs from normal tibial bone marrow or bone marrow collected 10 days postinjection of cancer cells were incubated with rabbit anti-mouse GPR84 (Affinity) for 30 min. Then, the samples were incubated with a secondary antibody conjugated with AF488 for another 30 min in the dark. The percentage of GPR84-positive BMMs was detected by a BD FACSVerse flow cytometer (BD Biosciences).

### Quantitative real-time PCR (qRT‒PCR)

To detect the mRNA expression of GPR84 in BMMs in vivo, BMMs were isolated from tibial bone marrow at the indicated timepoints after the injection of cancer cells. To examine the cell cycle biomarkers and osteoclastogenic biomarkers, cells were treated and collected as described above.

Total RNA was extracted with TRIzol Reagent (Invitrogen). RNA (0.5 μg) was reverse transcribed using the PrimeScript RT reagent Kit (TakaraBio) according to the manufacturer’s instructions. Two microliters of cDNA were used to detect the level of mRNA using quantitative PCR with the SYBR Premix Ex TaqTMII Kit (TakaraBio). GAPDH was used as a control for normalization. The sequences of the primers were as follows:

GAPDH (forward: 5′-GCATCTTCTTGTGCAGTGCC-3′ and reverse: 5′-TACGGCCAAATCCGTTCACA-3′), Gpr84 (forward: 5′-AACTGGGAACCTCAGTCTCCA-3′ and reverse: 5′-GCCCAACACAGACTCATGGTA-3′), Cenpa (forward: 5′-AGCTCCAGTGTAGGCTCTCA-3′ and reverse: 5′-TGCTCTTCTGCAGGGTCTTG-3′), Cdc20 (forward: 5′-GTTCGGGTAGCAGAACACCA-3′ and reverse: 5′- CAGATGTCGTCCATCTGGGG-3′), Cdk1 (forward: 5′-ACTCGGCCTCTAAGCTCCT-3′ and reverse: 5′-AGGTTACGACGGACCCTCTC-3′), Ccnd1 (forward: 5′-CAGCCCCAACAACTTCCTCT-3′ and reverse: 5′-CAGGGCCTTGACCGGG-3′), Ctsk (forward: 5′-CTGGCTGGGGTTATGTCTCAA-3′ and reverse: 5′-GGCTACGTCCTTACACACGAG-3′), and Oc-stamp (forward: 5′-ACTCACAGTCAAATATGACGCCT-3′ and reverse: 5′-GTAGATGACAGTCGTGGGGC-3′).

### Western blot analysis

Cells were lysed on ice for 30 min using lysis buffer and protease inhibitor cocktail. Total proteins were collected and subjected to SDS‒PAGE before transfer to polyvinylidene difluoride (PVDF) membranes. The membranes were blocked with 5% BSA. The samples were then incubated with antibodies against GPR84 (Affinity), ERK (Cell Signaling Technology), p-ERK (Thr202/Tyr204) (Cell Signaling Technology), JNK (Cell Signaling Technology), p-JNK (Thr183/Tyr185) (Cell Signaling Technology), p38 MAPK (Cell Signaling Technology), p-p38 MAPK (Thr180/Tyr182) (Cell Signaling Technology), STAT1 (Cell Signaling Technology), p-STAT1 (Tyr701) (Cell Signaling Technology), and β-actin (Affinity) followed by incubation with HRP-conjugated IgG (1:5000). The target proteins were visualized by the ChemiDoc XRS system (Bio-Rad).

### Cytochemistry and immunohistochemistry (IHC)

BMMs were acquired as described above. After cell adhesion, the samples were fixed with 4% PFA. After removing the fixative and washing extensively with PBS, the cells were permeabilized with 0.5% Triton X-100 for 15 min at 37 °C and blocked with 0.5% BSA for 1 h. Samples were incubated with rabbit anti-mouse GPR84 (Affinity) at a concentration of 1:100 overnight. Then, donkey anti-rabbit IgG conjugated with Cy3 (Jackson ImmunoResearch) was used to label GPR84.

For TRAP staining, the hindlimbs of the mice were removed at the time of sacrifice, and the tibias were fixed in 4% PFA and decalcified in 10% EDTA for 3 weeks. Tissues were embedded in paraffin and sectioned at a thickness of 10 μm. Decalcified tibia sections were stained with TRAP (Wako) following the manufacturer’s instructions.

### μCT analysis

For μCT analysis, a Bruker Skyscan 1174 X-ray microtomograph with an isotropic voxel size of 12.0 μm was used to image the tibias. Scans were conducted in 4% paraformaldehyde, and an X-ray tube potential of 50 kV and an intensity of 800 μA were used. For trabecular bone analysis, a 0.6 mm region beginning 0.1 mm below the growth plate to the 0.6 mm distance was contoured. 3D images were obtained from contoured 2D images using N-Recon. All images presented are representative of the respective groups.

### Statistical analysis

All data are representative of at least three experiments. Data are expressed as the mean ± SD. Differences between the results of two groups were evaluated using two-tailed Student’s t test. One-way ANOVA followed by a post hoc Dunnett’s test was used to determine the significance of the difference between the results from three or more groups. *p < 0.05, **p < 0.01 and ***p < 0.001 were regarded as significant.

## Results

### GPR84 is downregulated in OCPs after cancer bone metastasis

**Fig. 1:**
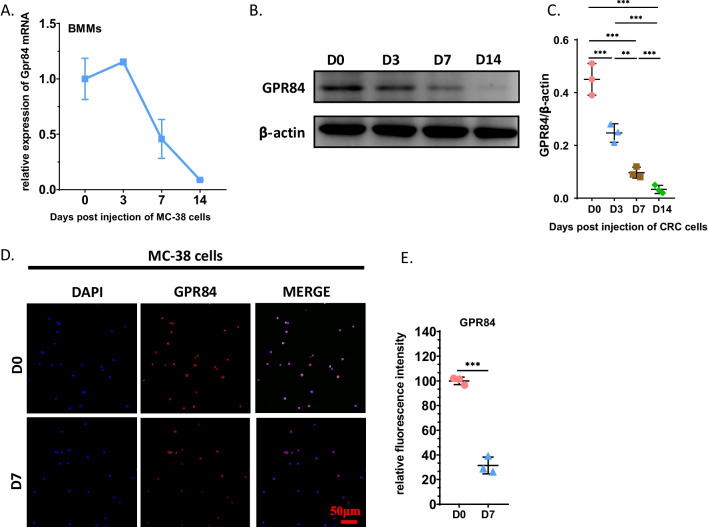
GPR84 downregulates in BMMs in bone metastatic model of CRC. **A** BMMs were isolated from bone marrow in tibias at indicated timepoints post-injection of MC-38 cells. The relative GPR84 levels were determined by qRT-PCR (*n* = 3). **B** and **C** The expression levels of GPR84 in BMMs isolated from bone marrow at indicated timepoints post-injection of MC-38 cells were determined by western blotting assay **B** and the protein level of GPR84 was normalized to β-actin (**C**) (*n* = 3). **D** and **E** Representative immunofluorescence images of GPR84 in BMMs isolated from bone marrow in mice at D0 or D7 post-injection of MC-38 cells (**D**) and quantification of relative expression of GPR84 (**E**) (*n* = 3). Bar represents 50 μm. Significant differences are indicated as **p < 0.01 or ***p < 0.001

There are few studies on whether the expression of GPR84 in BMMs can be regulated by CRC cells. We first detected the mRNA levels of GPR84 in BMMs at each timepoint after injection of CRC cells into the tibial bone marrow. During bone metastasis of CRC, the expression of GPR84 was highest in normal BMMs, and then the expression level decreased gradually with the progression of cancer bone metastasis (Fig. 1A). Consistently, the protein level of GPR84 in BMMs also decreased (Fig. 1B and C). Immunofluorescence demonstrated that the expression of GPR84 in isolated BMMs from bone marrow in bone metastases of CRC at 7 DPI was significantly downregulated compared with that in normal bone marrow (Fig. 1D–E). These data suggested that both the mRNA and protein levels of GPR84 in BMMs are downregulated during bone metastasis of CRC from the early stage.

### Overexpression of GPR84 directly inhibits osteoclast formation

**Fig. 2:**
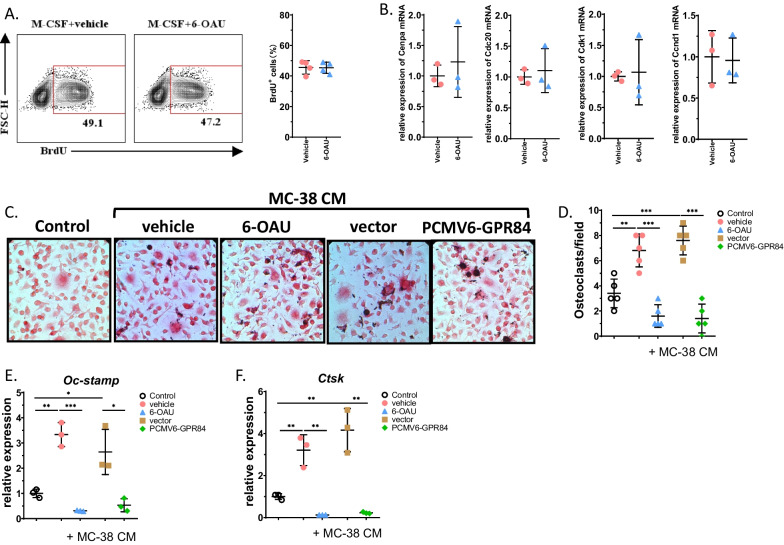
Overexpression of GPR84 directly inhibits osteoclast formation. **A** Representative image of flow cytometry (left) and quantification (right) of BrdU incorporation in BMMs treated by 6-OAU (200 nM) or vehicle in the presence of M-CSF (50 ng/mL) (*n* = 4). **B** BMMs isolated from bone marrow were treated by 6-OAU (200 nM) or vehicle in the presence of M-CSF (50 ng/mL) and the relative expression of cell cycle biomarkers was determined by qRT-PCR (*n* = 3). **C**, **D** Representative TRAP stain images of BMMs treated with CM from MC-38, 6-OAU or transfected with PCMV6-GPR84 (**C**) and quantification of osteoclast number (**D**) (*n* = 5). Bar represents 50 μm. **E** BMMs were isolated from bone marrow treated with CM from MC-38, 6-OAU or transfected with PCMV6-GPR84. The relative OCSTAMP level was determined by qRT-PCR (*n* = 3). **F** BMMs were isolated from bone marrow treated with CM from MC-38, 6-OAU or transfected with PCMV6-GPR84. The relative Ctsk level was determined by qRT-PCR (*n* = 3). Significant differences are indicated as *p < 0.05, **p < 0.01 or ***p < 0.001

To understand how GPR84 impacts BMMs in the tumor microenvironment, BMMs were sorted from normal bone marrow and cultured in the presence of M-CSF. Then, an agonist of GPR84, 6-OAU, was utilized to treat BMMs. Proliferation assays indicated that BrdU incorporation into BMMs treated with 6-OAU or vehicle showed no significant difference (Fig. 2A). Similarly, when detecting cell cycle biomarkers, qRT‒PCR analysis showed that the relative mRNA expression of Cenpa, Cdc20, Cdk1 and Ccnd1 was not significantly changed in the 6-OAU-treated group compared with the control group (Fig. 2B). These findings demonstrated that GPR84 does not directly regulate the proliferative capacity of BMMs.

To test the direct effects of GPR84 on osteoclastogenesis in the tumor microenvironment, BMMs were treated with 6-OAU or transfected with GPR84-overexpressing plasmids in the presence of CM collected from MC-38 cells as well as RANKL and M-CSF. The TRAP staining results suggested that BMMs treated with 6-OAU or transfected with GPR84-overexpressing plasmids showed significantly lower TRAP activity and fewer osteoclasts (Fig. 2C, D). The mRNA levels of two classic osteoclastogenic biomarkers, OCSTAMP and CTSK, were also markedly downregulated in the 6-OAU group and the GPR84-overexpressing plasmid group (Fig. 2E and F). These data indicated that GPR84 could directly inhibit osteoclastogenesis in the tumor microenvironment.

### GPR84 prevents osteoclast formation by inhibiting MAPK pathways

**Fig. 3:**
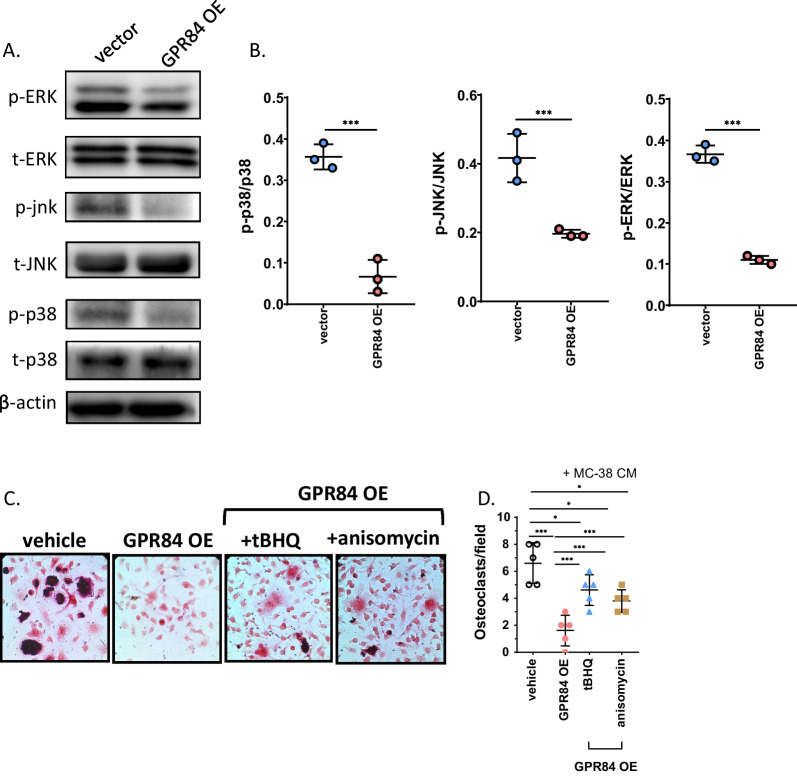
GPR84 prevents osteoclast formation through inhibiting MAPK pathways. **A** and **B** BMMs isolated from bone marrow were cultured in CM from MC-38 cells and transfected with GPR84-overexpressing vectors (GPR84 OE) or vectors. The protein levels of ERK, JNK, p38 MAPK and their phosphorylation of these proteins were determined by western blotting assay and the protein levels were normalized to β-actin (**A**) and quantification of the protein levels normalized to β-actin (**B**) (*n* = 3). **C**, **D** Representative TRAP stain images of BMMs were cultured in CM from MC-38 cells, then the cells were transfected with or without GPR84-overexpressing vectors or blank vectors (**C**) and quantification of osteoclast number (**D**) (*n* = 5). Bar represents 50 μm. Significant differences are indicated as *p < 0.05, **p < 0.01 or ***p < 0.001

To understand the underlying mechanism by which GPR84 inhibits osteoclastogenesis in the tumor microenvironment, we detected several intracellular pathways associated with osteoclastogenesis that are regulated by GPR84. Interestingly, activation of the MAPK pathway in BMMs was inhibited after overexpressing GPR84 when cultured in CM derived from MC-38 cells (Fig. 3A and B). In addition, activation of the ERK, JNK or p38 MAPK pathways using agonists efficiently reversed the inhibitory effect of overexpressing GPR84 on osteoclast formation in the tumor microenvironment (Fig. 3C, D). These data indicated that GPR84 regulated osteoclastogenic differentiation partly through MAPK pathways.

### IL-11 derived from cancer cells partially inhibits the expression of GPR84 in early OCPs

**Fig. 4:**
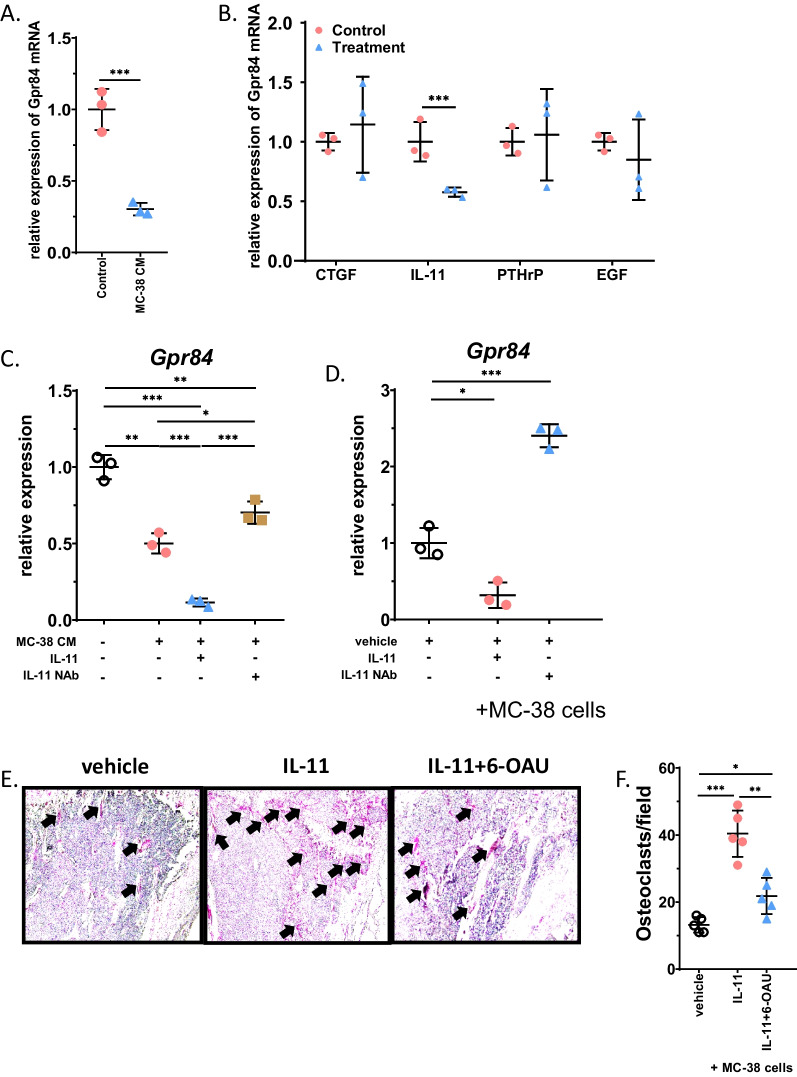
IL-11 derived from cancer cells partially inhibits the expression of GPR84 in BMMs. **A** BMMs were isolated from bone marrow and cultured in CM from MC-38. The relative GPR84 level was determined by qRT-PCR (*n* = 3). **B** BMMs were isolated from bone marrow and treated by recombinant CTGF, IL-11, PTHrP or EGF. The relative GPR84 level was determined by qRT-PCR (*n* = 3). **C** BMMs were isolated from bone marrow and cultured in CM from MC-38 with treatment of IL-11 or IL-11 neutralizing antibody. The relative GPR84 level was determined by qRT-PCR (*n* = 3). **D** BMMs were isolated from bone marrow in mice post-injection of MC-38 cells at D7 with administration of IL-11 or IL-11 neutralizing antibody. The relative GPR84 level was determined by qRT-PCR (*n* = 3). **E** and **F** Representative TRAP stain images of tibias post-injection of MC-38 cells at D21 with administration of IL-11 with or without 6-OAU (**E**) and quantification of osteoclast number (**F**) (*n* = 5). Bar represents 50 μm. Significant differences are indicated as *p < 0.05, **p < 0.01 or ***p < 0.001

To investigate whether the expression of GPR84 can be directly regulated by cancer cells, BMMs were cultured in CM from MC-38 cells for 48 h. The mRNA expression of GPR84 was significantly downregulated in CM-treated BMMs (Fig. 4A). Next, we explored which component(s) in CM could regulate the expression of GPR84. BMMs were stimulated by four common components secreted by cancer cells as reported by previous studies [11, 21], including EGF, IL-11, CTGF and PTHrP. qRT‒PCR analysis showed that only IL-11 downregulated the expression of GPR84 in BMMs (Fig. 4B). Then, we investigated whether blocking IL-11 in CM can regulate the expression of GPR84 in BMMs. Consistently, the expression of GPR84 was significantly downregulated after culture in CM. However, the addition of recombinant IL-11 to the CM enhanced the effect of CM on the expression of GPR84. Interestingly, the effect of CM on the level of GPR84 was significantly reversed in the presence of IL-11 NAb (Fig. 4C), indicating that blocking the endogenous IL-11 secreted by cancer cells could regulate the expression of GPR84. Next, we investigated whether GPR84 can also be regulated by IL-11 in vivo during cancer bone metastasis. IL-11 or IL-11 NAb were injected intraperitoneally in a bone metastasis model. The expression of GPR84 in BMMs isolated from bone marrow at 7 DPI was significantly higher in the IL-11 NAb-treated group than in the control group and IL-11-treated group (Fig. 4D). These data indicated that IL-11 could regulate the expression of GPR84 both in vitro and in vivo.

To investigate whether GPR84 can inhibit osteoclastogenesis caused by IL-11 during cancer bone metastasis, IL-11 was injected into bone marrow with or without 6-OAU in bone metastasis models of BC. TRAP staining results showed that the number of osteoclasts increased after the administration of IL-11; however, activation of GPR84 using 6-OAU efficiently inhibited the effects of IL-11 on osteoclastogenesis (Fig. 4E, F). Together, these results indicated that activating GPR84 could prevent osteoclast formation in cancer bone metastasis.

### STAT1 mediates the regulation of GPR84 by IL-11

**Fig. 5:**
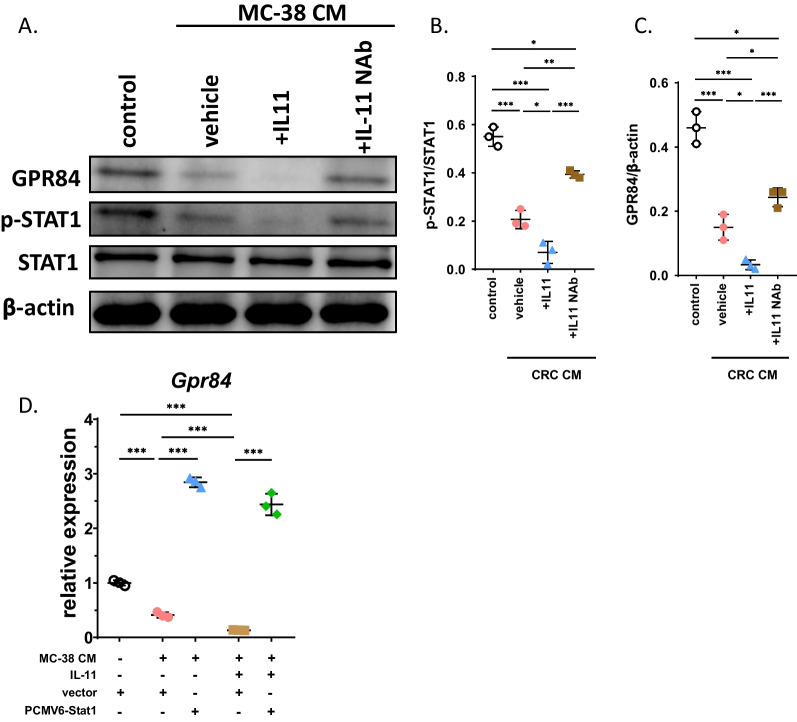
STAT1 mediates the regulation of IL-11 on GPR84. **A**–**C** BMMs isolated from bone marrow were cultured in CM and treated with IL-11 or IL-11 neutralizing antibody. The protein levels of STAT1 and phosphorylated STAT1 and GPR84 were determined by western blotting assay (**A**) and quantification the protein levels were normalized to β-actin (**B** and **C**) (*n* = 3). **D** BMMs isolated from bone marrow were cultured in CM and treated with IL-11 or transfected with PCMV6-STAT1. The relative GPR84 level was determined by qRT-PCR (*n* = 3). Significant differences are indicated as *p < 0.05, **p < 0.01 or ***p < 0.001

The STAT pathway was reported to be downstream of IL-11 [22]. Interestingly, STAT1 was predicted to be the transcription factor of GPR84. Thus, we investigated whether the activation of STAT1 in BMMs could be regulated by CM or IL-11. BMMs were cultured in CM from MC-38 cells with or without administration of IL-11 or its NAb. The Western blot results indicated that phosphorylated STAT1 was upregulated in the CM-treated group, and the administration of IL-11 enhanced this effect. However, STAT1 activation was inhibited after treatment with the IL-11 NAb (Fig. 5A–C). Furthermore, we explored whether the protein level of GPR84 in BMMs proceeded in an opposite manner to STAT1 activation (Fig. 5A–C). These results demonstrated that STAT1 in BMMs can be activated in the tumor microenvironment as well as IL-11, and blockage of IL-11 could alleviate this effect.

To investigate whether STAT1 mediated the IL-11-mediated regulation of GPR84 expression, BMMs were transfected with plasmids overexpressing STAT1. The mRNA level of GPR84 was significantly decreased after culture in CM. Moreover, treatment with IL-11 further downregulated the expression of GPR84. However, transfection with STAT1-overexpressing plasmids reversed this effect (Fig. 5D). These data indicated that IL-11 could regulate the expression of GPR84 by inactivating STAT1.

### Activation of GPR84 restores bone volume during cancer bone metastasis

**Fig. 6:**
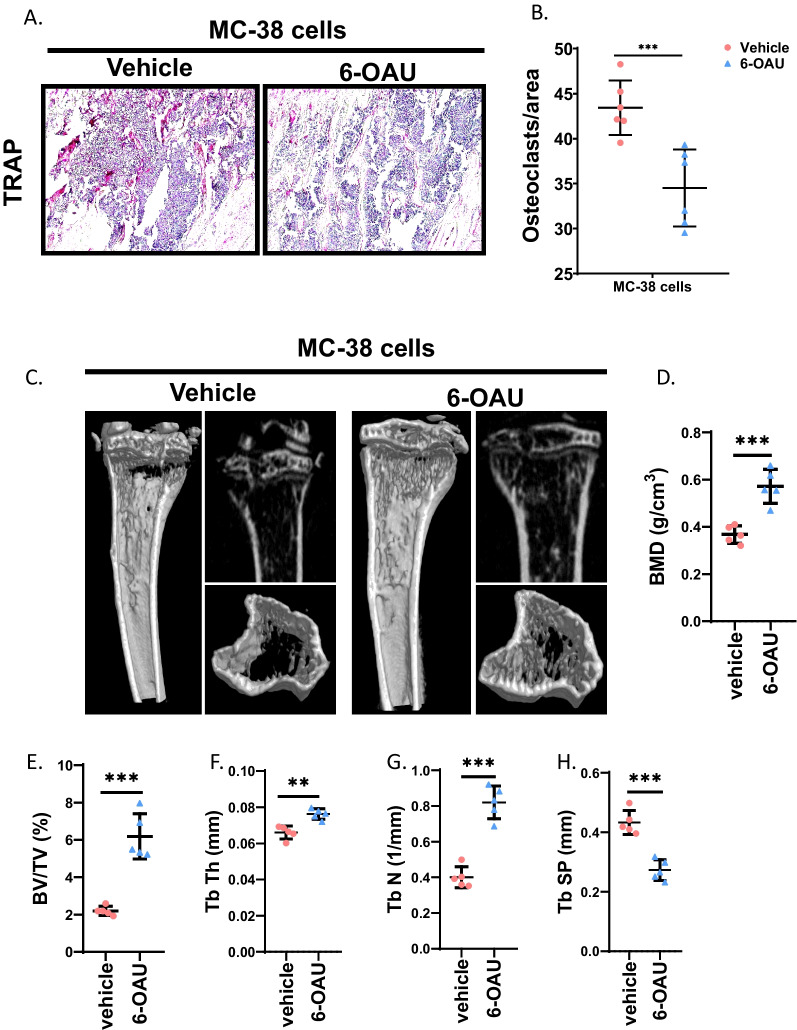
Activation of GPR84 restores the bone volume in cancer bone metastasis. **A** and **B** Representative TRAP stain images of tibias post-injection of cancer cells at D21 with administration of 6-OAU (**A**) and quantification of osteoclast number (**B**) (*n* = 6). Bar represents 50 μm. **C** and **D** Representative μCT of tibias.at 3 weeks post-injection of MC-38 cells (**C**) and quantification of bone mineral density (**D**) (*n* = 5). **E**–**H** Quantification of trabecular bone volume fraction (BV/TV) (**E**), trabecular thickness (Tb Th) (**F**), trabecular number (Tb. N) (**G**), trabecular separation (Tb Sp) (**H**) in (**C**) (*n* = 5). Significant differences are indicated as **p < 0.01 or ***p < 0.001

To explore whether GPR84 could be a potential therapeutic target for tumor-induced osteolysis, 6-OAU was administered to the bone marrow after injection of MC-38 cells. The TRAP staining results indicated that the number of osteoclasts was significantly decreased in 6-OAU-treated mice compared to that in control mice (Fig. 6A and B). The μCT results further revealed that 6-OAU significantly increased bone volume in week 3 (Fig. 6C). In addition, quantitative analysis showed that the bone mineral density (BMD) and trabecular bone volume fraction (BV/TV) increased significantly in the 6-OAU-treated group compared with the control groups (Fig. 6D and E). Moreover, quantitative analysis of the trabecular and cortical bone of the proximal tibia showed that the trabecular thickness (Tb. Th) was significantly increased in the 6-OAU-treated group compared with the control group. The trabecular number (Tb. N) also significantly increased in the 6-OAU-treated group compared with the control group (Fig. 6F and H). These data indicated that 6-OAU could prevent osteoclastogenesis and restore bone resorption caused by cancer bone metastasis.

## Discussion

CRC is classified as an osteolytic tumor. After bone metastasis, osteoclasts or their progenitors are abnormally activated to cause bone resorption. Several important pathways, such as the MAPK, NF-κB, and PI3K/AKT pathways, promote osteoclast formation. During this process, external stimuli are critical to commit progenitors to osteoclastogenic fate through paired ligand receptors. It has been well established that secreta derived from cancer cells regulate monocyte/macrophage commitment to an osteoclastogenic fate. Many secreting factors, receptors or intracellular proteins are involved in this process, which consists of a complex regulatory network. The most important signals come from the ligand and receptor pair RANKL/RANK; however, many other ligands/receptors are involved in amplifying or inhibiting this signal. It was reported that CD137-CD137L can promote the migration and osteoclastogenesis of monocytes/macrophages to facilitate the colonization and growth of cancer cells in bone [23]. TGF-β/TGFbr1 is required for the effects of cancer cells on NFATc1 nuclear accumulation and osteoclastogenesis [24]. CTGF/integrin αvβ3 signaling is also positively associated with osteoclastogenesis in BC and prostate cancer bone metastasis [25]. GPRs are an important receptor family for the transduction of external signals into cells. The function of GPR members in osteoclastogenesis is distinct. GPR4 overexpression in osteoblasts leads to the production of RANKL and promotes osteoclast formation in an acidic environment [26]. In contrast, GPR40/GPR120 inhibit osteoclastogenesis [27]. Similarly, the downregulation of GPR65 mediated by miR-7062-5p promotes osteoclast formation [17]. In this study, we found that GPR84 also inhibited osteoclastogenesis in CRC bone metastasis. In the natural progression of cancer bone metastasis, the expression of GPR84 was downregulated in the tumor microenvironment; however, activation of GPR84 through 6-OAU, a potent agonist, inhibited osteolysis. At a minimum, one important osteoclastogenesis-related pathway, i.e., the MAPK can be negatively regulated by GPR84, which is a conclusion that has been supported by another study [19]. The regulation of GPR84 by multiple pathways may explain the underlying reason for the potent inhibitory effect of GPR84 on osteoclastogenesis.

IL-11 was reported to be an important inflammatory cytokine for bone metastasis of cancer cells. For example, miR-124 prevented BC bone metastasis by targeting IL-11 [28]. Moreover, IL-11 mediated bone metastasis by regulating the vicious osteolytic cycle of bone resorption and thus promoted tumor growth [29], implying that IL-11 may regulate osteoclast formation. Liang et al. reported that IL-11 facilitated osteoclastogenesis in a RANKL-independent manner by activating the STAT3 pathway [11]. Herein, we found that GPR84 was a new target of IL-11 that stimulates osteoclast formation in the tumor microenvironment, providing a novel mechanism in IL-11-mediated osteoclastogenesis. In addition, IL-11-STAT3 signaling was reported to be closely associated with cancer metastasis [11, 30, 31]. However, we demonstrated that STAT1 is another downstream target of IL-11 in the regulation of cancer metastasis. STAT1 plays a distinct role with STAT3 in IL-11-mediated osteoclastogenesis in the tumor microenvironment. Other studies have found that STAT1 has an antitumor effect, as it can inhibit T-cell exhaustion and myeloid-derived suppressor cell accumulation in head and neck squamous cell carcinoma [32]. The invasion and metastasis of gastric cancer can be promoted upon inactivation of IFNgamma/STAT1 signaling [33]. Moreover, STAT1 can also inhibit angiogenesis in tumors [34]. In this study, we found that STAT1 can inhibit bone metastasis of CRC, providing new insight into the STAT1-mediated inhibition of cancer metastasis. Similarly, a previous study reported that increased phosphorylation of STAT1 could inhibit OCSTAMP and DCSTAMP, two important osteoclast-associated genes [35], which supports our findings. However, we also noticed that blockade of IL-11 can only partially reverse the downregulated expression of GPR84 caused by cancer CM, implying that some other unknown components in CM inhibited the level of GPR84, which should be further investigated.

## Data Availability

All data generated or analyzed during this study are included in this published article and its supplementary information files.

## Abbreviations

CRC: Colorectal cancer
OC: Osteoclast
OCP: Osteoclast precursors
GPR: G protein-coupled receptors
wk: Week
GM: Growth medium
CM: Condition medium
RBC: Red blood cells
BMM: Bone marrow-derived monocytes/macrophages
t-BHQ: Tert-butylhydroquinone
TRAP: Tartrate-resistant acid phosphatase
DPI: Days post-injection
BMD: Bone mineral density
BV/TV: Trabecular bone volume fraction
Tb. Th: Trabecular thickness
Tb. N: Trabecular number
